# Longitudinal monitoring of laboratory markers characterizes hospitalized and ambulatory COVID-19 patients

**DOI:** 10.1038/s41598-021-93950-x

**Published:** 2021-07-14

**Authors:** Thirumalaisamy P. Velavan, Salih Kuk, Le Thi Kieu Linh, Carlos Lamsfus Calle, Albert Lalremruata, Srinivas Reddy Pallerla, Andrea Kreidenweiss, Jana Held, Meral Esen, Julian Gabor, Eva Maria Neurohr, Parichehr Shamsrizi, Anahita Fathi, Erwin Biecker, Christoph P. Berg, Michael Ramharter, Marylyn Martina Addo, Benno Kreuels, Peter G. Kremsner

**Affiliations:** 1 Institute of Tropical Medicine, University of Tübingen, Wilhelmstrasse 27, 72074 Tübingen, Germany; 2 Vietnamese-German Center for Medical Research, Hanoi, Vietnam; 3 Centre de Recherches Médicales de Lambaréné, Lambaréné, Gabon; 4 Department of Tropical Medicine, Bernhard Nocht Institute for Tropical Medicine, Hamburg, Germany; 5 I Department of Medicine, University Medical Center Hamburg-Eppendorf, Hamburg, Germany; 6 German Center for Infection Research (DZIF), Partner Site Hamburg-Lübeck-Borstel-Riems, Brunswick, Germany; 7 Division of Infectious Diseases, First Department of Medicine, University Medical Center Hamburg-Eppendorf, Hamburg, Germany; 8 Department of Clinical Immunology of Infectious Diseases, Bernhard Nocht Institute for Tropical Medicine, Hamburg, Germany; 9 Zollernalb Hospital Balingen, Tübinger Straße 30, 72336 Balingen, Germany; 10 Department of Internal Medicine I, University of Tübingen, Tübingen, Germany; 11 Department of Medicine, College of Medicine, Blantyre, Malawi

**Keywords:** Diagnostic markers, Predictive markers, Prognostic markers

## Abstract

Early detection of severe forms of COVID-19 is absolutely essential for timely triage of patients. We longitudinally followed-up two well-characterized patient groups, hospitalized moderate to severe (n = 26), and ambulatory mild COVID-19 patients (n = 16) at home quarantine. Human D-dimer, C-reactive protein (CRP), ferritin, cardiac troponin I, interleukin-6 (IL-6) levels were measured on day 1, day 7, day 14 and day 28. All hospitalized patients were SARS-CoV-2 positive on admission, while all ambulatory patients were SARS-CoV-2 positive at recruitment. Hospitalized patients had higher D-dimer, CRP and ferritin, cardiac troponin I and IL-6 levels than ambulatory patients (*p* < 0.001, *p* < 0.001, *p* = 0.016, *p* = 0.035, *p* = 0.002 respectively). Hospitalized patients experienced significant decreases in CRP, ferritin and IL-6 levels from admission to recovery (*p* < 0.001, *p* = 0.025, and *p* = 0.001 respectively). Cardiac troponin I levels were high during the acute phase in both hospitalized and ambulatory patients, indicating a potential myocardial injury. In summary, D-dimer, CRP, ferritin, cardiac troponin I, IL-6 are predictive laboratory markers and can largely determine the clinical course of COVID-19, in particular the prognosis of critically ill COVID-19 patients.

## Introduction

The outbreak of the novel coronavirus SARS-CoV-2 causing COVID-19 was first reported in Wuhan, China, and has rapidly spread around the world, causing a global pandemic. Individual risk factors recognized to be associated with a more severe clinical outcome of COVID-19 are age above 65 years and underlying conditions, such as obesity, chronic obstructive lung disease and diabetes^1^. COVID-19 symptoms may include fever (83–99%), cough (59–82%), fatigue (44–70%), anorexia (40–84%), shortness of breath (31–40%), myalgia (11–35%) and other symptoms^2^.

The clinical course of COVID-19 can be classified in four stages. A presymptomatic and/or asymptomatic phase that lasts a few days is characterized as a laboratory-confirmed diagnosis in an infected person without overt symptoms^2,3^. Mild COVID-19 is characterized by fever, cough, fatigue, myalgia but without signs of viral pneumonia or hypoxia, whereas a moderate manifestation is characterized by pneumonia. Critical illness can occur as acute respiratory distress syndrome (ARDS), sepsis or septic shock^2^. Infection may progress to severe COVID-19 with dyspnoea and severe chest symptoms, with significant changes visible by chest x-rays and other imaging techniques, including ground glass abnormalities, patchy consolidation, alveolar exudates and interlobular involvement, eventually indicating deterioration^4^. While most infected persons have a mild illness and recover from the disease without requiring hospitalization, about 20% of patients with moderate or severe COVID-19 are hospitalized for additional supportive care^5^. Recently, models have calculated predictive scores, based on clinical parameters in order to determine the clinical course. Equally, several clinical and laboratory markers have been identified that may modulate the clinical course of COVID-19^1^.

COVID-19 is associated with varying degrees of coagulopathy, and the initial coagulopathy of COVID-19 has been characterized by increased D-dimers and fibrinogen or fibrin degradation products^6^. D-dimer concentrations greater than 0.5 μg/mL are associated with a severe course and D-dimer concentrations > 1 μg/mL are the strongest independent predictor of mortality^7^. C-reactive protein (CRP) levels have been shown to correlate with lung lesions and severe presentation in the early stages of COVID-19^8^. CRP levels are elevated in COVID-19 patients, and survivors have been shown to have mean CRP values of approximately 40 mg/L, while non-survivors had mean values of 125 mg/L, indicating a strong correlation with disease severity and unfavorable prognosis^1,9^. In addition, severe COVID-19 with immune-mediated inflammation, especially the development of a cytokine storm, is associated with the release of a large amount of pro-inflammatory cytokines, including TNF-alpha, IP-10, IL-1, IL-2 and IL-6^10,11^. Elevated ferritin levels due to secondary hemophagocytic lymphohistiocytosis (sHLH)^12^ and the cytokine storm syndrome have been reported in severe COVID-19 patients^11^. Hyperferritinemia is considered an independent risk factor in critically ill COVID-19 patients^12^. An increase in cardiac troponin indicates myocardial injury, and elevated cardiac troponin I (cTNI) levels associated with heart arrhythmia and death are commonly observed in severe COVID-19 patients^13^ and are an independent predictor of clinical outcome in critically ill COVID-19 patients^1^.

As treatment in intensive care units (ICU) is a major challenge, an early prognosis of severe forms of COVID-19 is absolutely essential. We investigated serum D-dimers, CRP, ferritin, cardiac troponin I, IL-6 levels during the acute phase of infection (SARS-CoV-2 RNA positive) and were monitored longitudinally in hospitalized patients with moderate to severe COVID-19 and in mild ambulatory COVID-19 patients in home quarantine.

## Results

### Baseline characteristics of COVID-19 patients

The baseline patient characteristics of the COVID-19 patients are summarized in Table 1. The mean number of days from first PCR positivity to recruitment defined as day 1 is 2.2 and the median number of days is 2 in hospitalized patients. In addition, individuals who tested positive by the health department were invited to participate in the ambulatory study. All ambulatory patients were recruited within 4 days of the onset of symptoms and illness along with PCR positivity. PCR positivity was also repeated on the day of recruitment. The mean number of days from initial PCR positivity to recruitment defined as day 1 was 3.7 and the median was 4 days in ambulatory patients. Of the 26 hospitalized patients examined, the median age was 62 years (range 21–91), 62% were male. Of the 16 examined ambulatory patients the median age was 43 years (range 18–63), 44% were male. Hospitalized patients were older than ambulatory patients (*p* < 0.001). None of the patients were pregnant. All hospitalized patients were SARS-CoV-2-positive on admission and most were negative on day 7 (except 5 positives) and all on day 14 (with the exception of one patient). The ambulatory patients had experienced a quick recovery and were SARS-CoV-2 negative by day 14 (with the exception of two patients).

**Table 1:** Baseline characteristics of hospitalized and ambulatory COVID-19 patients.

COVID-19 patient characteristics	Hospitalized (n = 26)	Ambulatory (n = 16)	*p*-value
Median age in years (range)	62 (21–91)	43 (18–63)	< 0.001
Male (n) (%)	16 (62%)	7 (44%)	0.272
Median blood pressure (systolic/diastolic)	133/78	143/92	0.073
Median respiratory rate (breaths/min.) (SD)	20 (± 3.2)	18 (± 3.1)	0.095
Median heart rate (beats per minute) (SD)	81 (± 10.7)	71 (± 18.0)	0.105
**Geometric mean (range min.–max.)**			
Leucocytes (n/µL)	4219 (3230–12,300)	NA	
Neutrophils (%)	55 (36–82)	NA	
Lymphocytes (%)	19 (8–49)	NA	
Monocytes (%)	7 (2–14)	NA	
Eosinophils (%)	1 (0–4)	NA	
Basophils (%)	0.5 (0.1–1)	NA	
Erythrocytes (Mio/µL)	4 (3–6)	NA	
Hematocrit (%)	33 (28–47)	NA	
Hb (g/dL)	11.5 (8–16)	NA	
Thrombocytes (1000/µL)	182 (105–572)	NA	
*Fibrinogen (mg/dL)	21 (211–768)	NA	
Procalcitonin (ng/mL)	0.07 (0.01–3)	NA	
Creatine kinase (U/L)	46 (19–2468)	NA	
AST (U/L)	18 (16–748)	NA	
ALT (U/L)	20 (9–401)	NA	
LDH (U/L)	181 (153–899)	NA	

NA: not available, as mild COVID-19 were ambulatory patients without clinical complications; min.—minimum; max.—maximum; *Fibrinogen levels not available for one hospitalized patient.

### D-dimers

**Figure 1:**
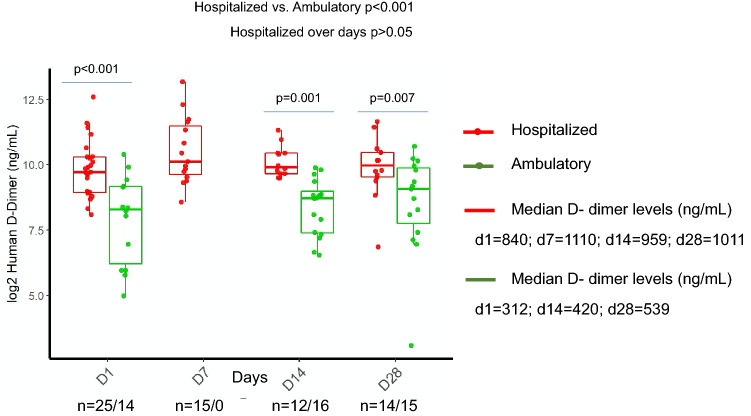
Longitudinal monitoring of D-dimers in hospitalized and ambulatory COVID-19 patients. Figure was created using ggplot2 package version 3.3.0. supported on R program software^38^.

The distribution of D-dimers was compared longitudinally and between the study groups (Fig. 1). The hospitalized patients had higher D-dimer values than the ambulatory patients (*p* < 0.001). Median D-dimer values were consistent throughout the clinical course in hospitalized and ambulatory patients. D-dimer values on admission, d14 and d28 were significantly higher in hospitalized compared to ambulatory patients (day 1: 840 ng/mL vs. 312 ng/mL, *p* < 0.001; day 14: 959 ng/mL vs. 420 ng/mL, *p* = 0.001; day 28: 1011 ng/mL vs. 539 ng/mL, *p* = 0.007).

### C-reactive protein

**Figure 2:**
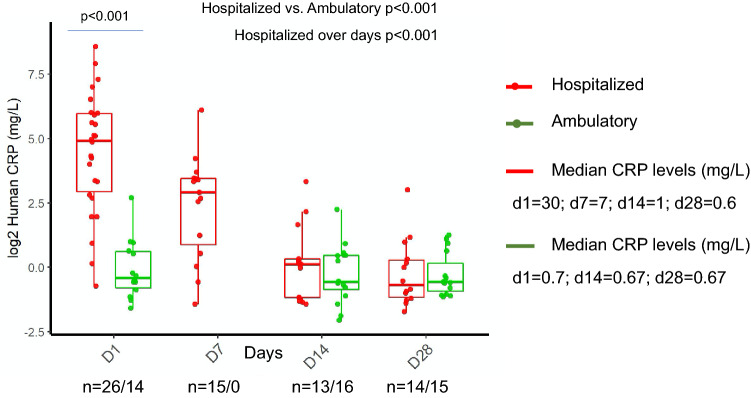
Longitudinal monitoring of C-reactive protein in hospitalized and ambulatory COVID-19 patients. Figure was created using ggplot2 package version 3.3.0. supported on R program software^38^.

Hospitalized patients had higher CRP levels than ambulatory patients (Fig. 2, *p* < 0.001). A significant difference was observed between hospitalized and ambulatory patients on day 1 (*p* < 0.001). Hospitalized patients had a significant decrease in CRP values from admission to recovery (*p* < 0.001), while ambulatory patients showed consistent CRP values.

### Ferritin

**Figure 3:**
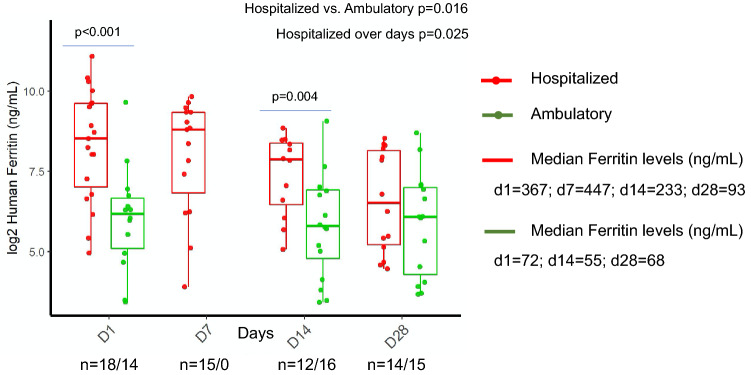
Longitudinal monitoring of ferritin levels in hospitalized and ambulatory COVID-19 patients. Figure was created using ggplot2 package version 3.3.0. supported on R program software^38^.

Hospitalized patients had higher ferritin levels than ambulatory patients (Fig. 3, *p* = 0.016). In hospitalized patients, median ferritin levels were high on day 1 (367 ng/mL) and day 7 (447 ng/mL) and decreased on day 14 (233 ng/mL) and day 28 (93 ng/mL) during recovery. Ferritin levels decreased significantly in hospitalized patients during the clinical course (*p* = 0.025) and the post-hoc test showed a significant difference from day 1 to day 28 (*p* = 0.007). In ambulatory patients, median ferritin levels were consistent throughout the clinical course. A significant difference was observed between hospitalized and ambulatory patients on day 1 (*p* < 0.001) and day 14 (*p* = 0.004).

### Cardiac troponin I

**Figure 4:**
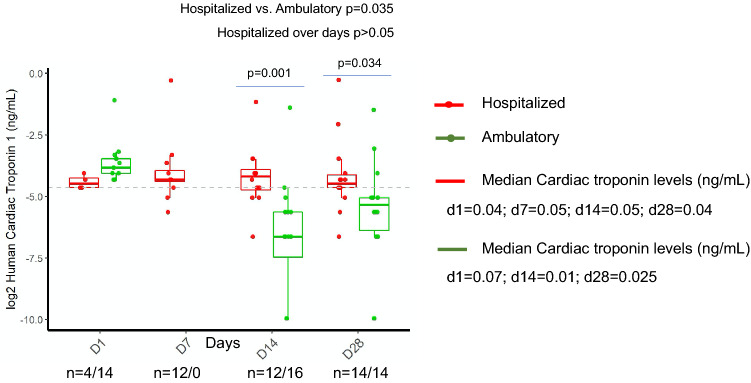
Longitudinal monitoring of Cardiac troponin I in hospitalized and ambulatory COVID-19 patients. Figure was created using ggplot2 package version 3.3.0. supported on R program software^38^.

Hospitalized patients had higher cardiac troponin I levels than ambulatory patients (Fig. 4, *p* = 0.035). A clear indication of possible heart muscle damage by SARS-CoV-2 during the acute phase of the infection can be observed in both hospitalized and ambulatory patients (Fig. 4). In hospitalized patients, median cardiac troponin I levels were consistent from admission to recovery. In ambulatory patients, median cardiac troponin I levels increased during the acute phase (day 1: 0.07 ng/mL) and then decreased during the recovery phase, declining to lower levels to hospitalized patients (day 14: 0.05 vs. 0.01 ng/mL, *p* = 0.001 and day 28: 0.04 vs. 0.025, *p* = 0.034). Fatal cardiac events due to myocardial injury caused by SARS-CoV-2 are predicted, which is reflected by a laboratory reference value > 0.04 ng/mL. On day 1, all hospitalized patients and outpatients had high levels of cardiac troponin I, above the threshold (log2 of 0.04 is -4.643, Fig. 4), indicating a myocardial injury. The threshold levels were crossed for day 1 in 4/4 hospitalized (≧ 0.04) and in 14/14 (≧ 0.04) outpatients. On day 7, 10/12 (≧ 0.04) hospitalized had high levels of cardiac troponin I; whereas for day 14, 9/12 hospitalized and 2/16 outpatients were above the threshold (≧ 0.04). Similar results were observed on day 28, when 11/14 hospitalized patients were above the threshold (≧ 0.04) compared to 3/14 in outpatients.

### Interleukin 6

**Figure 5:**
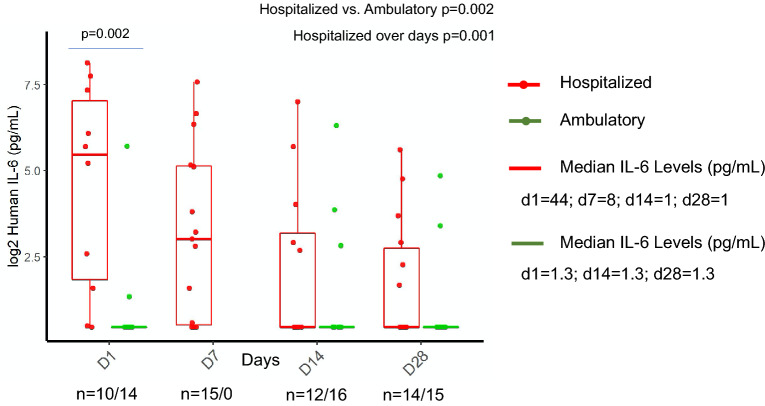
Longitudinal monitoring of interleukin-6 in hospitalized and ambulatory COVID-19 patients. Figure was created using ggplot2 package version 3.3.0. supported on R program software^38^.

IL-6 levels were higher in hospitalized patients compared to ambulatory patients (*p* = 0.002, Fig. 5). A significant difference was observed between hospitalized and ambulatory patients on day 1 (*p* = 0.002). Hospitalized patients had a significant decrease in IL-6 levels from admission to recovery (*p* = 0.001), while ambulatory patients did not show elevated levels and were below the detection limit.

## Discussion

Early detection of severe forms of COVID-19 is absolutely essential for timely triage of patients. In this study with two well-characterized patient groups, moderate to severe COVID-19 patients (hospitalized) and mild COVID-19 patients (ambulatory), we demonstrate that D-dimer, CRP, ferritin, cardiac troponin I and IL-6 levels can facilitate the differentiation of disease severity and further support clinical decision making.

Pathophysiology, clinical manifestations and underlying mechanisms for complications during COVID-19 yet remain unclear. Several studies have reported on correlations of abnormal coagulation parameters with poor prognosis, characterized by elevations in fibrinogen and D-dimer levels^7,14–16^. In this study, we observed that hospitalized patients had significantly higher D-dimer levels than ambulatory patients and approximately three times higher levels in hospitalized patients. Elevated D-dimer levels are more likely to reflect pulmonary intravascular coagulopathy^17^ as well as systemic immunothrombosis due to a hypercoagulable state as reflected in conventional tests of haemostasis and global tests of haemostasis^18–20^, during critical illness. While D-dimer is elevated, other classic criteria of DIC such as hypofibrinogenemia, markedly prolonged PT and thrombocytopenia are not present (according to ISTH criteria for DIC) and fibrinolysis is reduced^20^. Most patients do not develop disseminated intravascular coagulation, although a small subgroup of patients do. D-dimer elevation is capable to predict worse outcomes, but their explicit role warrants further investigations with respect to subsequent anticoagulant therapy^21,22^. Despite adequate thromboprophylaxis, recent evidences indicate that patients admitted to intensive care units develop deep vein thrombosis and pulmonary embolism^23,24^, suggesting a distinct role of coagulopathy in the pathogenesis of COVID-19^16^.

In addition to COVID-19 associated coagulopathy, a parallel rise in CRP levels was observed in severely ill patients. During early stages of the disease, CRP levels were positively correlated with lung lesions and severe presentation^8^ and these findings corroborate our results. CRP values were significantly distributed between hospitalized and ambulatory cases. In hospitalized patients, a clear pattern of significant decrease in CRP levels can be observed from admission to recovery. Studies have shown that CRP levels correlate positively with disease severity and progression^25–27^. Since CRP production is induced by tissue destruction and cytokines^28^, critically ill COVID-19 patients show the phenomenon of an aggressive inflammatory reaction, called "cytokine storm syndrome’’. No elevated CRP levels were observed in ambulatory patients. Especially during mild viral respiratory infections, CRP does not increase significantly. Another probable reason is that the inflammatory cytokines involved in the defense against the pathogen were not aggravated in mild patients.

Scientists have reasoned that longitudinal monitoring of ferritin during hospitalization may help to predict the progression of COVID-19 towards a worse clinical prognosis^29^. In this study, hospitalized patients had higher ferritin levels than ambulatory patients (425 ng/mL vs. 72 ng/mL), with ferritin levels decreasing on day 14 and day 28 during recovery, while in ambulatory patient ferritin levels remained constantly lower throughout the clinical course. Elevated ferritin levels due to secondary hemophagocytic lymphohistiocytosis and cytokine storm syndrome have been reported in severe COVID-19 patients^1,30,31^. Severe COVID-19 patients display immune dysregulation and hyperferritinemia is a key indicator for dysregulated hyperinflammatory immune response in critically ill patients^32^ and may also be considered as a pathogenic mediator.

Myocardial injury, detected by elevated troponin levels, has been associated with mortality in critically ill COVID-19 patients^13,33,34^. In this study, a clear indication of heart muscle damage by SARS-CoV-2 during the acute infection is observed both in mild ambulatory and hospitalized patients. In the hospitalized group, cardiac troponin I remained constant throughout the clinical course, whereas in ambulatory patients, median cardiac troponin I levels were high during the acute phase and subsequently declined. Elevated cardiac troponin I levels indicate prognostic information beyond ECG parameters, clinical signs and symptoms. Fatal cardiac events due to myocardial injury caused by SARS-CoV-2 are predicted, which is reflected by a laboratory reference value > 0.04 ng/mL. Myocardial injury may play a significant role in predicting poor outcomes in patients without a known history of chronic coronary syndromes^35^. However, a very recent study shows abundant evidence of heart damage in COVID-19 patients^36^. This retrospective study that examined 41 autopsies of COVID-19 patients concluded that cardiac tissues of the 30/41 cases harboured the SARS-CoV-2 virus in their hearts, while others have experienced inflammation of the sac surrounding the heart^36^.

In hospitalized patients, IL-6 concentrations were consistently high between day 1 and day 7. As almost all hospitalized patients recovering from COVID-19 showed viral negativity on day 14, IL-6 levels decreased between day 14 and day 28. No IL-6 mediated inflammatory cytokine responses were observed in ambulatory patients.

Although very well characterized patients were used in the study to investigate laboratory parameters over time, a larger sample size would be an obvious advantage to obtain more data for conclusive statements. Also, the number of days between onset of symptoms and viral positivity was not documented, thus day 1 of recruitment, in particular may reflect a different phase of the disease. Despite this limitation, the complete clinical course significantly differed between the two study cohorts. Taken together, while the age and concomitant comorbidities of COVID-19 patients largely determine the clinical course of COVID-19, D-dimer, CRP, ferritin, cardiac troponin I and IL-6 may act as predictors for severe COVID-19.

## Methods

### Ethics approval

Informed written consent was obtained from all study participants after detailed explanation of the study at the time of blood and serum sampling. Ethics approval was obtained from the Ethics Commission of the Medical Faculty of the Eberhard-Karls University and the University Hospital of Tübingen, and the Ethics Committee of the Ärztekammer Hamburg for two hydroxychloroquine trials (EudraCT Number 2020-001224-33 and EudraCT Number 2020-001512-26). All methods were performed in accordance with the relevant national guidelines and international regulations.

### Consent for publication

All authors agreed with the results and conclusions. All authors consented this version of the manuscript to be published.

### Study participants and sampling

A total of 354 hospitalized patients were screened for inclusion in the hospitalized group and 26 of those who met the inclusion criterion were enrolled. Although many hospitalized patients were eligible for recruitment, only 7% of the eligible patients were included in the hospitalized group, as many patients did not consent to the recruitment. Ambulatory patients included 16 of those who met the inclusion criterion. Demographic parameters such as age, gender, and additional information on concurrent morbidities were documented. The baseline characteristics of all recruited patients are summarized in Table 1. The majority of the hospitalized patients received supplemental oxygen due to respiratory distress, while none of the ambulatory patients reported breathing difficulties. Both hospitalized and ambulatory patients were followed longitudinally. Serum samples were collected from hospitalized patients on days 1, 7, 14 and 28 and from ambulatory patients on days 1, 14 and 28. Oropharyngeal samples were collected on the day of recruitment and immediately tested for SARS-CoV-2 RNA positivity, and serum samples were aliquoted and frozen at − 70 °C until further use. A detailed description of the inclusion and exclusion criteria for hospitalized COVID-19 patients (EudraCT-Number 2020-001224-33; https://www.clinicaltrialsregister.eu/ctr-search/trial/2020-001224-33/DE) and for ambulatory COVID-19 patients (EudraCT Number: 2020 001512-26; https://www.clinicaltrialsregister.eu/ctr-search/trial/2020-001512-26/DE) are recorded in the EU Register of Clinical Studies.

### RNA extraction and SARS-CoV-2 diagnosis

Viral RNA was extracted from oropharyngeal samples using the QIAamp Viral RNA Mini Kit (Qiagen, Hilden, Germany) according to the manufacturer's instructions. Subsequently, RealStar® SARS-CoV-2 real-time PCR targeting the S gene of SARS-CoV-2 (Altona Diagnostics, Hamburg, Germany) was performed according to the manufacturer's protocol. An in vitro transcribed RNA of the SARS-CoV-2 'S' gene was integrated in each run to determine the number of viral copies with respective Ct values. All hospitalized patients with moderate to severe COVID-19 (n = 26) and mild ambulatory patients in home quarantine (n = 16) were SARS-CoV-2 RNA positive on the day of admission and recruitment respectively.

### D-dimer, CRP, ferritin, cardiac troponin I, IL-6 concentrations

The concentrations of serum D-dimer, C-reactive protein, ferritin, cardiac troponin I and interleukin 6 were determined with a human D-dimer ELISA kit, a human CRP ELISA kit, a human ferritin ELISA kit, a human cardiac troponin I ELISA kit and an IL-6 ELISA kit (Abcam, Cambridge, UK) measured on a PHOmo microplate reader (Autobio Diagnostics, Zhengzhou, China) according to the manufacturer's instructions. For hospitalized patients, day 1, day 7, day 14 and day 28 samples were used, while for ambulatory patients, day 1, day 14 and day 28 were used. The detection limits for D-dimer, C-reactive protein, ferritin, cardiac troponin I and interleukin 6 were 2.36 ng/mL, 5.36 pg/mL, 5 ng/mL, 4.4 pg/mL and 3 pg/mL respectively.

### Statistical analysis

All statistical analysis was computed using R program software version 3.6.3. All data was log2 transformed for analysis and for better graphical illustration. Multiple comparisons for days and different study groups were computed using a mixed effect linear model following ANOVA. This mixed model uses a compound symmetry covariance matrix and is fitted using Restricted Maximum Likelihood (REML). Variation analysis was corrected for multiple comparison by controlling the False Discovery Rate (FDR) using the two-stage step-up method of Benjamini, Kireger and Yekutieli^37^. Figures were created using ggplot2 package version 3.3.0. supported on R program software^38^.

## Data Availability

All related data supporting the results reported in the article is available within the manuscript.
